# Study Protocol for the Fukushima Health Management Survey

**DOI:** 10.2188/jea.JE20120105

**Published:** 2012-09-05

**Authors:** Seiji Yasumura, Mitsuaki Hosoya, Shunichi Yamashita, Kenji Kamiya, Masafumi Abe, Makoto Akashi, Kazunori Kodama, Kotaro Ozasa

**Affiliations:** 1 Radiation Medical Science Center for the Fukushima Health Management Survey, Fukushima, Japan; 1 福島県立医科大学放射線医学県民健康管理センター; 2 Department of Public Health, School of Medicine, Fukushima Medical University, Fukushima, Japan; 2 福島県立医科大学医学部公衆衛生学講座; 3 Department of Pediatrics, School of Medicine, Fukushima Medical University, Fukushima, Japan; 3 福島県立医科大学医学部小児科学講座; 4 Research Institute for Radiation Biology and Medicine, Hiroshima University, Hiroshima, Japan; 4 広島大学原爆放射線医科学研究所; 5 Department of Diagnostic Pathology, School of Medicine, Fukushima Medical University, Fukushima, Japan; 5 福島県立医科大学医学部病理病態診断学; 6 National Institute of Radiological Sciences, Chiba, Japan; 6 放射線医学総合研究所; 7 The Radiation Effects Research Foundation, Hiroshima, Japan; 7 放射線影響研究所

**Keywords:** cohort study, radiation, disaster, thyroid gland, mental health

## Abstract

**Background:**

The accidents that occurred at the Fukushima Daiichi Nuclear Power Plant after the Great East Japan Earthquake on 11 March 2011 have resulted in long-term, ongoing anxiety among the residents of Fukushima, Japan. Soon after the disaster, Fukushima Prefecture launched the Fukushima Health Management Survey to investigate long-term low-dose radiation exposure caused by the accident. Fukushima Medical University took the lead in planning and implementing this survey. The primary purposes of this survey are to monitor the long-term health of residents, promote their future well-being, and confirm whether long-term low-dose radiation exposure has health effects. This report describes the rationale and implementation of the Fukushima Health Management Survey.

**Methods:**

This cohort study enrolled all people living in Fukushima Prefecture after the earthquake and comprises a basic survey and 4 detailed surveys. The basic survey is to estimate levels of external radiation exposure among all 2.05 million residents. It should be noted that internal radiation levels were estimated by Fukushima Prefecture using whole-body counters. The detailed surveys comprise a thyroid ultrasound examination for all Fukushima children aged 18 years or younger, a comprehensive health check for all residents from the evacuation zones, an assessment of mental health and lifestyles of all residents from the evacuation zones, and recording of all pregnancies and births among all women in the prefecture who were pregnant on 11 March. All data have been entered into a database and will be used to support the residents and analyze the health effects of radiation.

**Conclusions:**

The low response rate (<30%) to the basic survey complicates the estimation of health effects. There have been no cases of malignancy to date among 38 114 children who received thyroid ultrasound examinations. The importance of mental health care was revealed by the mental health and lifestyle survey and the pregnancy and birth survey. This long-term large-scale epidemiologic study is expected to provide valuable data in the investigation of the health effects of low-dose radiation and disaster-related stress.

## Abstract (ja)

**【背景】:**

2011年3月11日の東日本大震災に引き続いて起こった福島第一原子力発電所の事故により、福島県民は長期間にわたる不安状態に陥っている。福島県は、震災直後に、事故による慢性の低線量被ばくによる健康影響を評価するため「県民健康管理調査」を開始した。福島県立医科大学はこの調査の企画、及び、実施の中心となった。本調査の第一の目的は、長期にわたり住民の健康を評価すること、住民の将来の幸福に寄与すること、そして、慢性の低線量被ばくによる健康影響があるかどうかを検証することである。 本報告は、「県民健康管理調査」の理論的根拠と実施状況を報告するものである。

**【方法】:**

本コホート調査は地震後に福島に居住しているすべての住民が対象となっており、調査は基本調査と4つの詳細調査から成り立っている。基本調査は全住民205万人の外部被ばく線量を評価するものである。内部被ばく線量は、ホールボディカウンターを用いて福島県が評価することになった。詳細調査は、福島県の18歳以下のすべての子供を対象とした超音波検査による「甲状腺検査」、避難区域のすべての住民を対象とした「健康診査」、及び、避難区域のすべての住民を対象とした「こころの健康度と生活習慣に関する調査」、及び、県内において3月11日時点で妊娠していたすべての女性における妊娠と出産に関する「妊産婦調査」から成り立っている。すべてのデータはデータベースに集められ、住民の支援、及び、被ばくによる健康影響の分析のために用いられる。

**【結論】:**

基本調査は30%に満たない低い回答率のため、健康影響の評価は難しい可能性がある。甲状腺の超音波検査を現時点までに受診した38,114人の子供たちの中に、甲状腺がんは認められなかった。「こころの健康度と生活習慣に関する調査」及び、「妊産婦調査」により、こころの健康管理の重要性が明らかになった。この長期の大規模疫学調査は、低線量被ばくと災害関連のストレスによる健康影響の調査として価値あるデータを提供することが期待されている。

## INTRODUCTION

The Great East Japan Earthquake occurred at 2:46 PM on 11 March 2011. Later a tsunami hit the Tokyo Electric Power Company’s Fukushima Daiichi Nuclear Power Plant, causing a radiation hazard in Fukushima Prefecture. Due to the possible health impacts, the Fukushima prefectural government decided to conduct the Fukushima Health Management Survey to assist in the long-term health management of residents. The Radiation Medical Science Center for the Fukushima Health Management Survey was established in Fukushima Medical University to carry out the survey.^1^

Japan experienced atomic bombings in Hiroshima and Nagasaki in 1945. Acute radiation injuries were investigated by a joint Japan–US team, beginning in September of that year.^2^ In 1947, the Atomic Bomb Casualty Commission (ABCC) was established to investigate the health impacts on atomic-bomb (A-bomb) survivors. Later, a large-scale cohort study of the survivors was begun to investigate the long-term stochastic effects of radiation. The study used data from the 1950 Japan national census, which was conducted 5 years after the exposure. ABCC continued follow-up surveys until 1975, when it was succeeded by the Radiation Effects Research Foundation (RERF), which has continued the study until the present. The initial population size of survivors was 284 000 at the time of the census, and the established cohort—ie, the Life Span Study cohort—consisted of 120 000 individuals.^3^^–^^5^

In April 1986, the worst nuclear disaster in human history occurred at the Chernobyl Nuclear Power Plant. The accident released a large quantity of radioactive contamination into the atmosphere. The USSR Ministry of Health started the Russian National Medical and Dosimetric Registry in June the same year to register residents exposed to radiation. They also launched a program to evaluate health impacts of radiation exposure and later released partial results.^6^ However, an epidemiologic study, which is a direct, reliable method for evaluating long-term radiation effects on public health, was unfortunately not implemented soon enough after the accident.^7^ Although small-scale epidemiologic investigations^8^ have been conducted since around 1989, no investigation has recruited subjects for a comprehensive evaluation of health impacts.

The primary purposes of the Fukushima Health Management Survey are to monitor the long-term health of residents, promote their future well-being, and determine whether long-term low-dose radiation exposure has health effects. The ongoing basic survey was begun at the end of June (approximately 3 months after the accident) to estimate external exposure doses in Fukushima Prefecture at the time of the accident. In addition, we decided on sequential implementation of detailed surveys of forced evacuees who had lived in the evacuation zone—a government-designated area (radius, 20 km) around the nuclear power plant. This report describes the rationale and implementation of the Fukushima Health Management Survey, provides preliminary information on the participants, and reports some of the survey findings.

## METHODS

### Survey population

**Figure 1.**
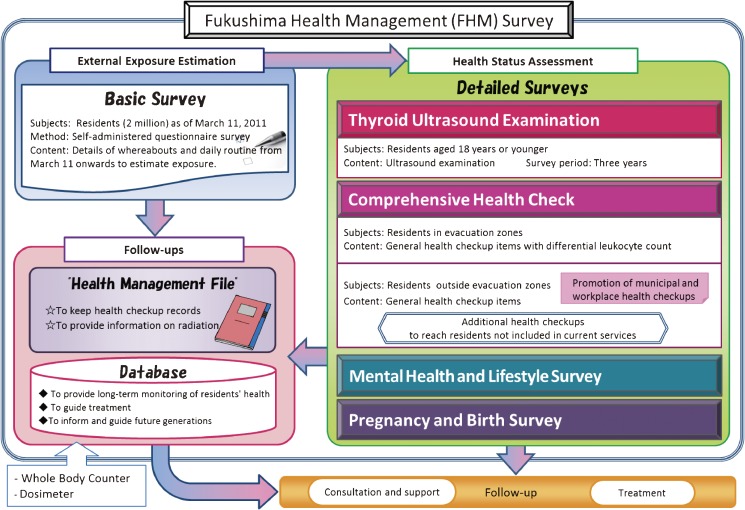
Framework of the Fukushima Health Management Survey

The Fukushima Health Management Survey consists of a basic survey and 4 detailed surveys, namely, the thyroid ultrasound examination, comprehensive health check, mental health and lifestyle survey, and pregnancy and birth survey (Figure 1). The target population of the basic survey is about 2.05 million. To be selected for the basic survey, individuals had to be either a registered resident of Fukushima Prefecture during the period of 11 March to 1 July (including those evacuated or transferred to residence registration in another prefecture); a resident of Fukushima Prefecture who was registered in another prefecture during the period of 11 March to 1 July; a resident of another prefecture who commuted to Fukushima Prefecture during the period of 11 March to 1 July; or a resident of another prefecture who temporarily stayed in Fukushima Prefecture during the period of 11 March to 25 March. The detailed surveys targeted selected people on the basis of the particular criteria used for each survey. External exposure dose, as indicated on the basic survey, and information from the detailed surveys will be linked and maintained in the database for later analysis.

### Basic survey

**Figure 2.**
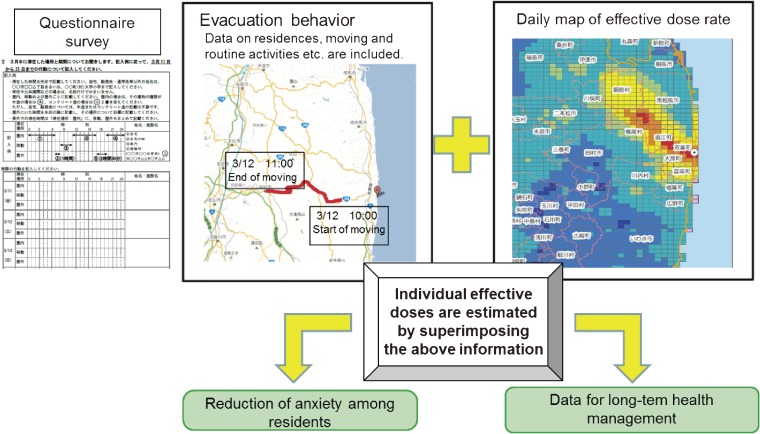
System for evaluating external radiation

Estimation of individual radiation dose, though costly and laborious, was essential for risk assessment in the Life Span Study of A-bomb survivors in Hiroshima and Nagasaki. Interviews were conducted from 1954 to 1965 to determine distance from the hypocenter and exposure conditions for each survivor.^5^ On the basis of that experience, we have implemented a rigorous system for estimating external exposure doses, with technical support from the National Institute of Radiological Sciences. We mailed self-administered questionnaires to collect information from residents on, among other variables, their dwelling place, places visited, length of time indoors and outdoors, and travelling time during the period from 11 March to 11 July, the period when atmospheric radiation dose was highest. The respondents were asked to mail back the questionnaires. To ensure that potential respondents understood that participation was noncompulsory, they received the following notification in the questionnaire cover letter: “Please note that participation in the survey is voluntary and that you will not be disadvantaged in any way if you decide not to participate.” This method of using respondent activities and location (“trail”) to evaluate external exposure dose is almost identical to the procedure used in the evaluation carried out after the Chernobyl accident.^9^ Individual external exposure was estimated based on a respondent’s trail, using the system for external exposure dose assessment developed by the National Institute of Radiological Sciences in Japan^10^ (Figure 2). It is important to note that internal radiation levels of Fukushima residents were measured separately by Fukushima Prefecture, using whole-body counters. The results showed that the estimated maximum internal exposure doses of Cs-134 and Cs-137 among 122 residents in Namie Town, Iitate Village, and the Yamakiya district of Kawamata Town were as low as less than 1 mSv.^11^

### Detailed surveys

#### Thyroid ultrasound examination

The Chernobyl accident revealed that thyroid cancer in children was increased by internal exposure to radioiodine.^12^^–^^14^ Thus, to ensure early identification and treatment of thyroid cancer in children, and their lifelong follow-up, we decided to perform thyroid ultrasound examinations on all children. Because the increase in thyroid cancer was reported to start 4 or 5 years after the Chernobyl accident,^15^ we expect no excess occurrence in the first 3 years in Japan. Therefore, assessment of current thyroid status will be completed within 3 years. Due to the importance of long-term follow-up of all children in the prefecture and the considerable anxiety of their parents, all children aged 18 years or younger in the prefecture will undergo ultrasound examination.

*Target*: All prefectural inhabitants aged between 0 and 18 years on 11 March 2011, ie, those born from 2 April 1992 to 1 April 2011, including evacuees living in other prefectures. The total population is approximately 360 000.

*Methods and criteria*: Thyroid ultrasound, the primary examination, is done using a device that (a) has a 10-MHz or higher frequency probe, (b) is able to save Digital Imaging and Communications in Medicine (DICOM) images, (c) has a color Doppler function, (d) is able to save moving images, and (e) is able to transfer saved data to media. Examiners are required to be a medical specialist of either the Japan Thyroid Association, the Japan Association of Endocrine Surgeons, the Japanese Society of Thyroid Surgery, or the Japan Society of Ultrasonics in Medicine (a body surface/general medical specialist); a pediatric specialist of the Japan Endocrine Society; or a laboratory technician (specializing in the body surface) from the Japanese Society of Sonographers.

When the primary examination reveals a nodule or cyst, a confirmatory examination is to be carried out at Fukushima Medical University Hospital or another hospital (certified by our expert committee) for advanced ultrasound examination. During the confirmatory examination, a detailed ultrasound, blood testing, urine analysis, and aspiration biopsy cytology are performed as necessary. Ultrasound devices used in the confirmatory examination must have a 18-MHz or higher frequency probe. Other device and personnel requirements are the same as those listed above for the primary examination. The confirmatory examination is to be done at institutions that employ suitably qualified examiners or pathologists. Specialists of the Japanese Society of Pathology are required for cytodiagnosis.

The following diagnostic criteria are used: A1, no nodule or cyst; A2, nodule less than 5.0 mm and/or cyst less than 20.1 mm; B, further examination necessary (nodule ≥5.0 mm and/or cyst ≥20.1 mm); and C, urgent need for further examination.

*Schedule*: The thyroid ultrasound examination is to be provided to all children for 2.5 years, from October 2011 to March 2014. From April 2014, children will undergo thyroid examination every 2 years until age 20 years and every 5 years after that. The target cohort will include those born before 1 April 2012. By April 2012, at least 1 institute was designated as an examination center in each of the 46 prefectures, to serve the approximately 20 000 evacuees living in other prefectures.

#### Comprehensive health check

Many evacuees from the government-designated evacuation zone were forced to change their lifestyle, diet, exercise, and other personal habits. Some could not receive adequate health checks, and some had anxieties about their health.^16^ The comprehensive health check attempts to review their health information, assess the incidence of various diseases, and improve their health status.

*Target*: The target groups were residents of all ages living in the evacuation zone specified by the government, ie, Hirono-machi, Naraha-machi, Tomioka-machi, Kawauchi-mura, Okuma-machi, Futaba-machi, Namie-machi, Kazurao-mura, Iitate-mura, Minamisoma City, and Tamura City. The residents of Yamakiya in Kawamata-machi, Namie-machi, and Iitate-mura also completed the comprehensive health check, even though that area is farther than 20 km away from the plant, because nuclear fallout spread northwest of the plant, and because their inclusion was required based on the results of the Basic Survey. The size of the target cohort is 210 189 as of 31 March 2011.

*Methods*: The following items have been added to the Special Health Checkup performed as part of the Municipal National Health Insurance system, which is performed among adults aged 40 years or older in the prefecture. For people who do not participate in the Special Health Checkup, a visiting comprehensive health check has been held a total of 104 times at 29 locations since January 2012. Children aged 0 to 15 years have received health checks at 102 pediatric medical institutions in the prefecture since January (153 pediatricians agreed to be registered to conduct the comprehensive health checks).

Comprehensive health checks have also been performed outside the prefecture, with the cooperation of the Japan Anti-Tuberculosis Association, at 827 member institutions of the Japan Municipal Hospital Association, the Japan National Health Insurance Clinics and Hospitals Association, the All-Japan Federation of Social Insurance Associations, and the Japan Red Cross Society. A total of 554 pediatric medical institutions also helped to conduct health checks for children aged 15 years or younger.

*Evaluation items*: In addition to assessing the effects of radiation, additional variables are specified according to age to assess health, prevent lifestyle-related diseases, and find or treat diseases at an early stage (Table 1). Individuals aged 16 years or older are evaluated according to items in the Specific Health Examination based on the Act on Assurance of Medical Care for Elderly People (Act No. 80, 1982). The examination includes measurements of height, weight, abdominal circumference/body mass index (BMI), blood pressure, aspartate aminotransferase (AST), alanine aminotransferase (ALT), γ-glutamyl transpeptidase (γ-GTP), triglyceride (TG), high-density lipoprotein-cholesterol (HDL-C), low-density lipoprotein-cholesterol (LDL-C), hemoglobin A1c (HbA1c), fasting blood glucose, and urine testing (protein and sugar). Additional items for assessment include blood count—red blood cell (RBC), hematocrit (Hct), hemoglobin (Hb), platelet count, white blood cell (WBC), and WBC count—serum creatinine (Cr), estimated glomerular filtration rate (eGFR), uric acid (UA), and urine testing for occult blood. The survey items for children aged 7 to 15 years are height, weight, blood pressure, RBC, Hct, HB, platelet count, WBC, and WBC count. Upon request, AST, ALT, γ-GTP, TG, HDL-C, LDL-C, HbA1c, serum Cr, eGFR, and UA are added. For children aged 0 to 6 years, height, weight, RBC, Hct, HB, platelet count, WBC, and WBC count are examined.

**Table 1.** Items included in comprehensive health check

Age, y	Items
0–6	Height, weight, blood count (RBC, Hct, HB, platelets, WBC, WBC count)
7–15	Height, weight, blood count (RBC, Hct, HB, platelets, WBC, WBC count)
	If requested by patient: Blood chemistry (AST, ALT, γ-GTP, TG, HDL-C, LDL-C, HbA1c, FBG, S-Cr, eGFR, UA)
≥16	Height, weight, abdominal circumference/BMI, BP
	Blood count (RBC, Hct, HB, Platelet, WBC count)
	Blood chemistry (AST, ALT, γ-GTP, TG, HDL-C, LDL-C, HbA1c, FBS, S-Cr, eGFR, UA)
	Urinary testing (protein, sugar, blood)

Abbreviations: γ-GTP, γ-glutamyl transpeptidase; ALT, alanine aminotransferase; AST, aspartate aminotransferase; BMI, body mass index; BP, blood pressure; eGFR, estimated glomerular filtration rate; FBG, fasting blood glucose; LDL-C, low-density lipoprotein-cholesterol; HbA1c, hemoglobin A1c; Hb, hemoglobin; Hct, hematocrit; HDL-C, high-density lipoprotein-cholesterol; RBC, red blood cells; S-Cr, serum creatinine; TG, triglyceride; UA, uric acid; WBC, white blood cells.

#### Mental health and lifestyle survey

Mental health disorders were an important long-term health effect of the Chernobyl accident.^17^^–^^27^ Deaths of close relatives, loss of home and property, and fearful experiences during the disaster resulted in psychological trauma for many residents of Fukushima Prefecture. Furthermore, some may have been mentally affected by evacuation, and others may have experienced anxiety regarding radiation exposure. To prevent excess mortality, it is essential to assess their mental health and lifestyle, prevent lifestyle-related diseases, and provide care as necessary.

*Target*: The target cohort was the same as that for the comprehensive health check, 210 189 people.

*Methods*: Questionnaires have been mailed since 18 January 2012.

*Survey items*: The survey items vary according to age category (there are 3 age categories for children and 1 for adults) but mainly ask about current mental and physical status, lifestyle (diet, sleep, smoking, alcohol, and exercise), activities during the last 6 months, and experience during the earthquake (Table 2). Parents of children aged 4 through 15 years are asked to evaluate their children using the Strength and Difficulties Questionnaire (SDQ).^28^ The K6 scale and PTSD Checklist Stressor-Specific Version (PCL) are self-administered for people 16 years or older.^29^^,^^30^

**Table 2.** Overview of mental health and lifestyle survey

Category	Age criteria	Mental health items	Method
Adults	Born before 1 April 1995 (ie, high school student on 11 March 2011)	K6 (Kessler, 2003); PCL (PTSD Checklist Stressor-Specific Version, Weathers, 1994)	Self-administered
Children (3)	Born after 2 April 1995 but before 1 April 1998 (ie, junior high school student on 11 March 2011)	SDQ (Strengths and Difficulties Questionnaire)	Partially self-administered
Children (2)	Born after 2 April 1998 but before 1 April 2004 (ie, primary school student on 11 March 2011)	SDQ	Completed by parents
Children (1)	Born after 2 April 2004 but before 11 March 2011 (ie, pre-primary school on 11 March 2011)	SDQ for children aged 4 years or older	Completed by parents

*Support after the survey*: Clinical psychologists and other specialists on the mental health support team offer telephone counseling as necessary based on answers to the questionnaires. When telephone counseling reveals a need for medical support, 93 registered doctors in medical institutions in Fukushima Prefecture are available for introduction. Further treatment is given by a specialist at Fukushima Medical University if necessary. These registered doctors are mainly psychiatrists or pediatricians who agreed to be registered and who attended a relevant seminar or are certified by Fukushima Medical University.

When telephone counseling reveals a need for care by a doctor specialized in radiation, a member of the Radiation Health Consultation Team, which consists of faculty at Fukushima Medical University, will be introduced.

#### Pregnancy and birth survey

The World Health Organization concluded in the “Health Effects of the Chernobyl Accident and Special Health Care Programmes, 2006”^7^ that the Chernobyl accident did not significantly increase child anomalies or fetal deaths related to radiation exposure. In Fukushima, no significant increase in induced abortion or miscarriage was observed after 11 March 2011; however, the nuclear accident required pregnant women to change clinics/hospitals, and many received insufficient antenatal care and faced difficulties in appropriately managing their personal health and that of their children.^31^ The pregnancy and birth survey aims to collect data that might improve obstetrical and perinatal care and support women who have, or plan to deliver, a baby in Fukushima Prefecture.

*Target*: Women who received Maternal and Child Health Handbooks from municipal offices in Fukushima Prefecture between 1 August 2010 and 31 July 2011 or those who had handbooks issued in other prefectures but delivered babies in Fukushima Prefecture on March 11 or later. The total number of women meeting these criteria is 15 954.

*Methods*: Questionnaires have been mailed out since 18 January 2012. Because many pregnant women evacuated to other prefectures, letters of request were sent to the Japan Society of Obstetrics and Gynecology and the Japan Association of Obstetricians and Gynecologists to request their help in enrolling pregnant evacuees in the survey.

*Survey items*: The survey included antenatal health and delivery records and mental health (Whooley’s 2-item case-finding instrument for depression).^32^

*Post-survey support*: Telephone and e-mail hot lines have been launched and midwives or public health nurses provide consultation services for health management, child care, and anxiety. Midwives and nurses from Fukushima Medical University also provide telephone consultations when further support is necessary. Depending on the problems revealed during telephone counseling, consultation can be arranged with the respondent’s regular obstetrician or one from Fukushima Medical University. For evacuees outside Fukushima Prefecture, support is provided by a survey team that includes university obstetricians.

This survey was approved by the ethical review committee of Fukushima Medical University (No. 1257, 1275, 1294, 1316, 1317, 1318, and 1319).

## DISCUSSION

The basic survey and all 4 detailed surveys in this large-scale cohort study were launched within 1 year of the accident.^33^ It was essential for the basic survey to begin as early as possible because its accuracy relies on participant memory. The health effects of radiation can be assessed by linking data on external exposure dose from the basic survey plus data on internal exposure level, as measured by whole-body counters, with detailed health data from the other 4 surveys, cancer registries, and vital statistics. There is limited scientific understanding of the human health impact of chronic low-dose exposure, and this study is thus of global importance.^34^ We should note that the regional cancer registry needs to be improved if we are to accurately assess changes in cancer incidence.

### Basic survey

As of 31 March 2012, responses were sent from 451 446 of 2 056 994 questionnaires, a response rate of 21.9%. This low rate is a critical problem, as the lack of external exposure dose data complicates health management and estimation of health impacts. The response rate exceeded 37% in the Soso district, which is close to the affected nuclear power plants, but was lower than 15% in Aizu and Minami-Aizu. The highest response rate by age was among those aged 60 to 69 years (60%), whereas the lowest rate was among those aged 20 to 29 years. Such variation in response rate may reflect differences in exposure awareness.

The low overall response rate suggests that the basic survey has not been recognized as the most important information source for health management. The aims of the survey appear in newspapers and regional newsletters, and in DVDs, posters, and leaflets that have been sent to kindergartens and schools. Participation in the basic survey is also encouraged at the institutions where thyroid or health examinations are held, and guidance on completing the survey questions is offered at temporary evacuee housing. Furthermore, a letter of request has been sent to nonrespondents, and municipal governments and various organizations and companies continue to raise awareness of the survey’s importance.

### Detailed surveys

#### Results of the thyroid ultrasound examination

In total, 38 114 (79.8%) of 47 766 people underwent thyroid examination by March 2012. With regard to diagnostic code, 99.5% were classified as A (A1 and A2), and most did not require a secondary examination; 186 (0.5%) were classified as B (14 of whom underwent secondary examination), but no malignancy was detected. None were classified as C.

#### Results of the comprehensive health check

As of 31 March 2012, the number of Fukushima Prefecture residents who underwent the health check was 48 530 of 149 159 (32.5%) children and adolescents aged 16 years or older and 13 557 of 19 303 (70.2%) of those aged 15 years or younger. For those living in other prefectures, the tentative figures were 8070 of 33 340 (24.2%) among those aged 16 or older and 4199 of 8387 (50.1%) among those aged 15 or younger. Overall, 74 556 of 210 189 (35.4%) participated in the health check. The data have been aggregated and will be announced when the examinations are finished.

#### Results of the mental health and lifestyle survey

As of 31 March 2012, the number of people who participated in the survey included 7713 of 11 717 children in category 1 on Table 2 (65.8%), 7377 of 11 791 in category 2 (62.6%), and 3330 of 6077 in category 3 (54.8%). Among adults, 70 193 of 180 604 (38.9%) participated in the survey. Overall, 88 613 answered the survey questionnaires (42.2%).

As part of telephone counseling, we attempt to identify people who need urgent support. To do so, we modified the cut-off values for the SDQ (16 was changed to 20), K6 (13 to 20), and PCL (44 to 70). In addition, answers to open-ended questions on the questionnaire were used to prioritize respondents. The results revealed that 4.7% of adults and 5.3% of children in category 1, 7.4% in category 2, and 6.4% in category 3 needed immediate support. The target of the telephone counseling service will be expanded after counseling is completed for the highest-priority group.

#### Results of the pregnancy and birth survey

Questionnaires were sent to 15 954 women; 8886 (55.7%) replied. A total of 1298 (14.6%) women gave affirmative answers to both depression items or were assessed as in need of support based on their free comments. They are offered telephone counseling from midwives, public health nurses, and nurses.

### Survey management and the International Expert Symposium

We are continuing to build the database. Although the results are preliminary, we have started reporting invaluable findings. Regarding the assessment of mental health, Boice stated that “the study of mental disorders [is] important and [should] be emphasized” because “the long-term psychological effects of such a disaster have not been well studied”.^35^

We face 4 important managerial issues. First, the call center in the Radiation Medical Science Center handles inquiries regarding details of the survey. Augmentation of this system is essential to provide residents with the necessary timely and ongoing support. Second, although the investigation will continue for 30 years, the road map from 2012 onwards has not been completed as of this writing. Mid- and long-term investigation schedules need to be completed soon. Third, residents should be given immediate feedback on the survey results and the scientific interpretation of the findings. Therefore, we urge the establishment of a research system that includes development of a database and an epidemiologic network for researchers. Last, it is critically important to improve risk communication, including appropriate and prompt information-sharing with residents regarding the aims, content, and results of the Fukushima Health Management Survey.

An expert committee was established for each survey. These committees meet almost every week and are attended by Japanese experts in each survey field. In addition, a managerial committee was formed on 19 May 2011. The members of the committee are Dr Masafumi Abe (Vice President) and Dr Seiji Yasumura (Professor, Department of Public Health) of Fukushima Medical University, who were asked to conduct the Fukushima Health Management Survey, and Dr Makoto Akashi (Executive Director, National Institute of Radiological Sciences), Dr Kazunori Kodama (Chief Scientist, The Radiation Effects Research Foundation), Dr Kenji Kamiya (Director, Research Institute for Radiation Biology and Medicine, Hiroshima University, Vice President, Fukushima Medical University), Dr Shunichi Yamashita (Vice President, Fukushima Medical University), Dr Hokuto Hoshi (Board Member, Fukushima Medical Association), Setsuo Sato (Former Director General, Social Health & Welfare Department, Fukushima Prefecture), and Hiroyuki Kanno (Present Director General, Social Health & Welfare Department, Fukushima Prefecture). Since the establishment of the committee, representatives of the Japanese Ministry of Economy, Trade and Industry; the Ministry of Education, Culture, Sports, Science and Technology; and the Ministry of Health, Labour and Welfare have attended the meetings as observers. A representative of the Ministry of Environment has participated since the fifth meeting, which was held on 25 January 2012.

We must establish a health system that can respond to the resident health care needs identified on the Fukushima Health Management Survey. Moreover, an international advisory board should be formed to increase support from international experts and verify that the survey is scientifically sound and credible.^36^ The Nippon Foundation, Sasakawa Memorial Health Foundation, and Fukushima Medical University organized The International Expert Symposium in Fukushima—Radiation and Health Risks, which was held in Fukushima on 11–12 September 2011. In its conclusions and recommendations, the authors write that “Initial plans for the Fukushima Health Management Survey were presented at the Symposium and were welcomed under the recognition that there is a critical need to develop organized community participation to express the collective concerns of the population as a whole”.^37^ This large-scale long-term epidemiologic study is likely to provide important data on the health effects of low-dose radiation and disaster-related stress. We need and desire ongoing concern and support from epidemiologists all over the world.

## ONLINE ONLY MATERIALS

Abstract in Japanese.

## APPENDIX

### The Fukushima Health Management Survey Group

*Chairpersons*: Shunichi Yamashita (Director, Radiation Medical Center for the Fukushima Health Management Survey), Kenji Kamiya (Vice Director, Radiation Medical Center for the Fukushima Health Management Survey), Seiji Yasumura (Vice Director, Radiation Medical Center for the Fukushima Health Management Survey), Mitsuaki Hosoya (Chief, Clinical Department, Radiation Medical Center for the Fukushima Health Management Survey), Masafumi Abe (Director General, Radiation Medical Center for the Fukushima Health Management Survey), Makoto Akashi (National Institute of Radiological Sciences), Kazunori Kodama, and Kotaro Ozasa (The Radiation Effects Research Foundation).

*Participating researchers and advisors*: Akira Ohtsuru, Akira Sakai, Makoto Miyazaki, Motomi Yokota, Tomoko Suzuki, Shiro Matsui, Koji Ohtani, Shinichi Suzuki, Shinichi Nakajima, Tsuyoshi Watanabe, Koichi Omori, Keiji Kanemitsu, Yukihiko Kawasaki, Keiichi Nakano, Toshihiko Fukushima, Takashi Matsuzuka, Sanae Midorikawa, Hidetsugu Yasuda, Mutsuo Yamahata, Yuji Imafuku, Hirosuke Sato, Hitoshi Suzuki, Yasuchika Takeishi, Shinichi Niwa, Hirooki Yabe, Hirofumi Mashiko, Yoko Nakayama, Michiko Yuki, Keiya Fujimori, Misao Ohta, Aya Goto, Hidenori Takahashi, Yasuhisa Nomura, Shun Yasuda, Satoshi Waguri, Suguri Ishida, Kazuhide Ogawa, Atsushi Kikuta, Yumiko Nakajima, Noboru Ohana, Yoshisada Shibata, and Hidetsugu Yasuda (Fukushima Medical University), Jiro Iwase (The University of Aizu), Kenichi Hata and Hiroshi Iwanami (Fukushima Medical Association), Akinobu Hata (Fukushima Prefectural Mental and Welfare Center), Keiichi Akahane, Gen Kobashi, Hiroshi Yasuda, and Shunsuke Yonai (National Institute of Radiological Sciences), Mitsuru Hisata (Sophia University), Yoko Kamio and Yuriko Suzuki (National Center of Neurology and Psychiatry), Suminori Akiba (Kagoshima University), Tomotaka Sobue (Osaka University), Norito Kawakami (The University of Tokyo), and Hiroshi Nishimoto and Akiko Shibata (National Cancer Center).
