# Supplementary material for: Study Protocol for the Fukushima Health Management Survey
Source: J Epidemiol. 2012 Sep 5;22(5):375–83. doi: 10.2188/jea.JE20120105 (PMC3798631; doi:10.2188/jea.JE20120105)
Supplement: Abstract in Japanese. [file je-22-375-s001.pdf]

福島県における県民健康管理調査プロトコール

安村誠司[1,2]、細矢光亮[1,3]、山下俊一[1]、神谷研二[1,4]、阿部正文[1,5]、明石真言[6]、児玉和紀[7]、小笹晃太郎[7]

[1]福島県立医科大学放射線医学県民健康管理センター、[2]福島県立医科大学医学部公衆衛生学講座、[3]福島県立医科大学医学部小児科学講座、[4]広島大学原爆放射線医科学研究所、[5]福島県立医科大学医学部病理病態診断学、[6]放射線医学総合研究所、[7]放射線影響研究所

【背景】2011 年 3 月 11 日の東日本大震災に引き続いて起こった福島第一原子力発電所の事故により、福島県民は長期間にわたる不安状態に陥っている。福島県は、震災直後に、事故による慢性の低線量被ばくによる健康影響を評価するため「県民健康管理調査」を開始した。福島県立医科大学はこの調査の企画、及び、実施の中心となった。本調査の第一の目的は、長期にわたり住民の健康を評価すること、住民の将来の幸福に寄与すること、そして、慢性の低線量被ばくによる健康影響があるかどうかを検証することである。
本報告は、「県民健康管理調査」の理論的根拠と実施状況を報告するものである。
【方法】本コホート調査は地震後に福島に居住しているすべての住民が対象となっており、調査は基本調査と 4 つの詳細調査から成り立っている。基本調査は全住民 205 万人の外部被ばく線量を評価するものである。内部被ばく線量は、ホールボディカウンターを用いて福島県が評価することになった。詳細調査は、福島県の 18 歳以下のすべての子供を対象とした超音波検査による「甲状腺検査」、避難区域のすべての住民を対象とした「健康診査」、及び、避難区域のすべての住民を対象とした「こころの健康度と生活習慣に関する調査」、及び、県内において 3 月 11 日時点で妊娠していたすべての女性における妊娠と出産に関する「妊産婦調査」から成り立っている。すべてのデータはデータベースに集められ、住民の支援、及び、被ばくによる健康影響の分析のために用いられる。
【結論】基本調査は 30%に満たない低い回答率のため、健康影響の評価は難しい可能性がある。甲状腺の超音波検査を現時点までに受診した 38,114 人の子供たちの中に、甲状腺がんは認められなかった。「こころの健康度と生活習慣に関する調査」及び、「妊産婦調査」により、こころの健康管理の重要性が明らかになった。この長期の大規模疫学調査は、低線量被ばくと災害関連のストレスによる健康影響の調査として価値あるデータを提供することが期待されている。

キーワード： コホート調査; 放射線; 災害; 甲状腺; メンタルヘルス
